# (2*E*,2′*E*)-1,1′-Bis(2,5-dimethyl-3-thien­yl)-3,3′-(*p*-phenyl­ene)diprop-2-en-1-one

**DOI:** 10.1107/S1600536809024581

**Published:** 2009-07-01

**Authors:** Abdullah Mohamed Asiri, Salman A. Khan, Seik Weng Ng

**Affiliations:** a Chemistry Department, Faculty of Science, King Abdul Aziz University, Jeddah, Saudi Arabia; b Department of Chemistry, University of Malaya, 50603 Kuala Lumpur, Malaysia

## Abstract

In the title bis-chalcone, C_24_H_22_O_2_S_2_, the –C(O)CH=CH–C_6_H_4_–CH=CHC(O)– portion is planar (r.m.s. deviation = 0.04 Å); one thienyl ring is aligned at 8.8 (1)° with respect to this fragment, whereas the other is aligned at 21.3 (1)°.

## Related literature

Chalcones possess anti-bacterial, anti-fungal and anti-inflammatory properties, see: Yarishkin *et al.* (2008 ▶); such properties are dramatically enhanced in bis-chalcones. For the crystal structures of some bis-chalcones, see: Harrison *et al.* (2007*a* ▶,*b* ▶,*c* ▶); Prajapati *et al.* (2008 ▶).
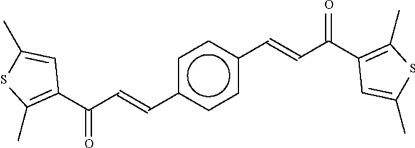


## Experimental

### 

#### Crystal data

C_24_H_22_O_2_S_2_
*M* *_r_* = 406.54
Monoclinic, 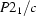

*a* = 15.6120 (12) Å
*b* = 7.5600 (6) Å
*c* = 18.2863 (14) Å
β = 111.305 (4)°
*V* = 2010.8 (3) Å^3^
*Z* = 4
Mo *K*α radiation
μ = 0.28 mm^−1^
*T* = 140 K
0.40 × 0.10 × 0.01 mm

#### Data collection

Bruker SMART APEX diffractometer
Absorption correction: multi-scan (*SADABS*; Sheldrick, 1996 ▶) *T*_min_ = 0.896, *T*_max_ = 0.997
10889 measured reflections
3537 independent reflections
2102 reflections with *I* > 2σ(*I*)
*R*_int_ = 0.098

#### Refinement

*R*[*F* ^2^ > 2σ(*F* ^2^)] = 0.052
*wR*(*F* ^2^) = 0.125
*S* = 0.97
3537 reflections
257 parameters
H-atom parameters constrained
Δρ_max_ = 0.27 e Å^−3^
Δρ_min_ = −0.28 e Å^−3^

### 

Data collection: *APEX2* (Bruker, 2008 ▶); cell refinement: *SAINT* (Bruker, 2008 ▶); data reduction: *SAINT*; program(s) used to solve structure: *SHELXS97* (Sheldrick, 2008 ▶); program(s) used to refine structure: *SHELXL97* (Sheldrick, 2008 ▶); molecular graphics: *X-SEED* (Barbour, 2001 ▶); software used to prepare material for publication: *publCIF* (Westrip, 2009 ▶).

## Supplementary Material

Crystal structure: contains datablocks global, I. DOI: 10.1107/S1600536809024581/tk2486sup1.cif

Structure factors: contains datablocks I. DOI: 10.1107/S1600536809024581/tk2486Isup2.hkl

Additional supplementary materials:  crystallographic information; 3D view; checkCIF report

## Figures and Tables

**Fig. 1.**
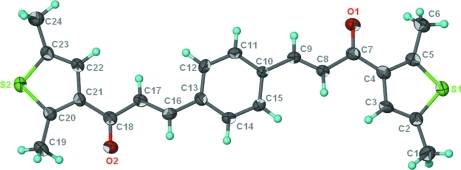
Thermal ellipsoid plot (Barbour, 2001) of C24H22O2S2 at the 70% probability level; hydrogen atoms are drawn as spheres of arbitrary radius.

## supplementary crystallographic information

### Experimental

A solution of 3-acetyl-2,5-dimethylthiophene (3.13 ml, 0.0074 mol) and terephthaldehyde (2 g, 0.0074 mol) in ethanolic sodium hydroxide (60%) was stirred for 20 h at room temperature. The solution was poured into ice-cold water and the pH of the mixture was adjusted to 2 by the addition of concentrated hydrochloric acid. The solid that separated was dissolved in dichloromethane and then washed with saturated sodium bicarbonate. The residual obtained upon removal of the solvent was recrystallized from a methanol–chloroform (1/1) mixture in 80% yield; m.p. 467–468 K.

### Refinement

Carbon-bound H-atoms were placed in calculated positions (C—H 0.95–0.98 Å) and were included in the refinement in the riding model approximation with *U*(H) fixed at 1.2–1.5*U*_eq_(C).

### Crystal data

**Table:**

C_24_H_22_O_2_S_2_	*F*(000) = 856
*M**_r_* = 406.54	*D*_x_ = 1.343 Mg m^−^^3^
Monoclinic, *P*2_1_/*c*	Mo *K*α radiation, λ = 0.71073 Å
Hall symbol: -P 2ybc	Cell parameters from 765 reflections
*a* = 15.6120 (12) Å	θ = 2.8–19.6°
*b* = 7.5600 (6) Å	µ = 0.28 mm^−^^1^
*c* = 18.2863 (14) Å	*T* = 140 K
β = 111.305 (4)°	Plate, yellow
*V* = 2010.8 (3) Å^3^	0.40 × 0.10 × 0.01 mm
*Z* = 4	

### Data collection

**Table:**

Bruker SMART APEX diffractometer	3537 independent reflections
Radiation source: fine-focus sealed tube	2102 reflections with *I* > 2σ(*I*)
graphite	*R*_int_ = 0.098
ω scans	θ_max_ = 25.0°, θ_min_ = 1.4°
Absorption correction: multi-scan (*SADABS*; Sheldrick, 1996)	*h* = −18→18
*T*_min_ = 0.896, *T*_max_ = 0.997	*k* = −8→8
10889 measured reflections	*l* = −21→21

### Refinement

**Table:**

Refinement on *F*^2^	Primary atom site location: structure-invariant direct methods
Least-squares matrix: full	Secondary atom site location: difference Fourier map
*R*[*F*^2^ > 2σ(*F*^2^)] = 0.052	Hydrogen site location: inferred from neighbouring sites
*wR*(*F*^2^) = 0.125	H-atom parameters constrained
*S* = 0.97	*w* = 1/[σ^2^(*F*_o_^2^) + (0.0496*P*)^2^] where *P* = (*F*_o_^2^ + 2*F*_c_^2^)/3
3537 reflections	(Δ/σ)_max_ = 0.001
257 parameters	Δρ_max_ = 0.27 e Å^−^^3^
0 restraints	Δρ_min_ = −0.28 e Å^−^^3^

### Fractional atomic coordinates and isotropic or equivalent isotropic displacement parameters (Å^2^)

**Table:**

	*x*	*y*	*z*	*U*_iso_*/*U*_eq_	
S1	0.05024 (6)	0.83736 (12)	0.17666 (5)	0.0311 (3)	
S2	0.83487 (6)	−0.22707 (12)	0.99588 (5)	0.0309 (3)	
O1	0.27593 (18)	0.9170 (3)	0.41549 (14)	0.0387 (7)	
O2	0.64980 (17)	−0.3043 (3)	0.73797 (14)	0.0377 (7)	
C1	0.0397 (3)	0.4820 (5)	0.1280 (2)	0.0389 (10)	
H1A	0.0806	0.3799	0.1347	0.058*	
H1B	0.0250	0.5316	0.0753	0.058*	
H1C	−0.0171	0.4443	0.1346	0.058*	
C2	0.0865 (2)	0.6196 (5)	0.1881 (2)	0.0284 (9)	
C3	0.1587 (2)	0.6012 (5)	0.2562 (2)	0.0295 (9)	
H3	0.1891	0.4915	0.2731	0.035*	
C4	0.1860 (2)	0.7622 (5)	0.3013 (2)	0.0264 (8)	
C5	0.1326 (2)	0.9034 (5)	0.2631 (2)	0.0272 (9)	
C6	0.1384 (3)	1.0950 (4)	0.2861 (2)	0.0358 (10)	
H6A	0.1373	1.1054	0.3391	0.054*	
H6B	0.0860	1.1591	0.2491	0.054*	
H6C	0.1958	1.1455	0.2851	0.054*	
C7	0.2590 (2)	0.7752 (5)	0.3798 (2)	0.0268 (8)	
C8	0.3077 (2)	0.6124 (4)	0.4165 (2)	0.0262 (9)	
H8	0.3004	0.5090	0.3853	0.031*	
C9	0.3615 (2)	0.6057 (4)	0.4917 (2)	0.0253 (8)	
H9	0.3682	0.7128	0.5205	0.030*	
C10	0.4115 (2)	0.4536 (4)	0.53522 (19)	0.0214 (8)	
C11	0.4739 (2)	0.4748 (5)	0.61117 (19)	0.0249 (8)	
H11	0.4832	0.5891	0.6344	0.030*	
C12	0.5228 (2)	0.3329 (5)	0.6538 (2)	0.0270 (9)	
H12	0.5657	0.3513	0.7056	0.032*	
C13	0.5099 (2)	0.1626 (4)	0.6218 (2)	0.0242 (8)	
C14	0.4463 (2)	0.1415 (5)	0.5455 (2)	0.0253 (8)	
H14	0.4363	0.0269	0.5227	0.030*	
C15	0.3978 (2)	0.2827 (4)	0.50260 (19)	0.0243 (8)	
H15	0.3549	0.2645	0.4508	0.029*	
C16	0.5607 (2)	0.0092 (5)	0.66412 (19)	0.0264 (9)	
H16	0.5522	−0.0976	0.6350	0.032*	
C17	0.6172 (2)	0.0010 (5)	0.7384 (2)	0.0272 (9)	
H17	0.6275	0.1049	0.7696	0.033*	
C18	0.6650 (2)	−0.1639 (5)	0.7744 (2)	0.0265 (9)	
C19	0.7270 (2)	−0.4819 (4)	0.8930 (2)	0.0322 (9)	
H19A	0.6629	−0.4868	0.8568	0.048*	
H19B	0.7321	−0.5351	0.9433	0.048*	
H19C	0.7658	−0.5473	0.8706	0.048*	
C20	0.7578 (2)	−0.2932 (4)	0.9058 (2)	0.0256 (8)	
C21	0.7327 (2)	−0.1511 (4)	0.8555 (2)	0.0229 (8)	
C22	0.7768 (2)	0.0090 (5)	0.8912 (2)	0.0274 (9)	
H22	0.7658	0.1194	0.8646	0.033*	
C23	0.8357 (2)	−0.0104 (4)	0.9663 (2)	0.0276 (9)	
C24	0.8943 (2)	0.1272 (5)	1.0203 (2)	0.0373 (10)	
H24A	0.8767	0.2445	0.9968	0.056*	
H24B	0.9591	0.1050	1.0287	0.056*	
H24C	0.8856	0.1224	1.0707	0.056*	

### Atomic displacement parameters (Å^2^)

**Table:**

	*U*^11^	*U*^22^	*U*^33^	*U*^12^	*U*^13^	*U*^23^
S1	0.0305 (5)	0.0267 (5)	0.0311 (6)	0.0034 (4)	0.0052 (4)	0.0043 (4)
S2	0.0339 (6)	0.0282 (6)	0.0260 (5)	0.0042 (4)	0.0055 (4)	0.0039 (4)
O1	0.0514 (17)	0.0216 (15)	0.0339 (15)	0.0018 (13)	0.0046 (13)	−0.0036 (13)
O2	0.0467 (17)	0.0211 (15)	0.0347 (16)	0.0028 (12)	0.0020 (13)	−0.0040 (13)
C1	0.040 (2)	0.034 (2)	0.035 (2)	0.002 (2)	0.0059 (19)	−0.006 (2)
C2	0.033 (2)	0.024 (2)	0.027 (2)	0.0048 (17)	0.0091 (18)	0.0001 (17)
C3	0.033 (2)	0.017 (2)	0.033 (2)	0.0030 (17)	0.0068 (18)	−0.0027 (17)
C4	0.030 (2)	0.023 (2)	0.029 (2)	0.0000 (17)	0.0133 (17)	0.0014 (18)
C5	0.028 (2)	0.023 (2)	0.031 (2)	−0.0021 (17)	0.0122 (17)	0.0001 (18)
C6	0.040 (2)	0.024 (2)	0.042 (2)	0.0027 (19)	0.012 (2)	0.0004 (19)
C7	0.032 (2)	0.022 (2)	0.027 (2)	−0.0032 (17)	0.0113 (17)	−0.0004 (18)
C8	0.031 (2)	0.018 (2)	0.026 (2)	0.0015 (16)	0.0057 (17)	−0.0010 (16)
C9	0.028 (2)	0.020 (2)	0.025 (2)	−0.0017 (16)	0.0062 (17)	−0.0029 (17)
C10	0.0234 (19)	0.018 (2)	0.0199 (19)	−0.0017 (15)	0.0046 (15)	0.0008 (15)
C11	0.028 (2)	0.020 (2)	0.026 (2)	−0.0042 (16)	0.0091 (16)	−0.0046 (17)
C12	0.027 (2)	0.029 (2)	0.0200 (19)	−0.0022 (17)	0.0021 (16)	0.0012 (17)
C13	0.0280 (19)	0.021 (2)	0.024 (2)	0.0010 (17)	0.0091 (16)	0.0021 (17)
C14	0.029 (2)	0.021 (2)	0.025 (2)	−0.0014 (16)	0.0085 (17)	−0.0040 (17)
C15	0.0236 (19)	0.024 (2)	0.0221 (19)	−0.0033 (16)	0.0041 (16)	−0.0017 (17)
C16	0.0256 (19)	0.023 (2)	0.028 (2)	−0.0041 (17)	0.0065 (17)	−0.0040 (17)
C17	0.032 (2)	0.020 (2)	0.028 (2)	0.0012 (17)	0.0101 (17)	−0.0016 (17)
C18	0.027 (2)	0.024 (2)	0.029 (2)	0.0014 (17)	0.0101 (17)	0.0018 (18)
C19	0.036 (2)	0.023 (2)	0.038 (2)	0.0039 (17)	0.0137 (18)	0.0047 (18)
C20	0.0252 (19)	0.022 (2)	0.029 (2)	0.0046 (16)	0.0082 (16)	0.0006 (17)
C21	0.0240 (18)	0.022 (2)	0.0227 (19)	0.0032 (16)	0.0081 (15)	0.0026 (17)
C22	0.032 (2)	0.021 (2)	0.027 (2)	0.0033 (17)	0.0079 (17)	0.0025 (17)
C23	0.028 (2)	0.024 (2)	0.030 (2)	0.0069 (17)	0.0090 (17)	0.0010 (18)
C24	0.036 (2)	0.029 (2)	0.040 (2)	−0.0009 (18)	0.0046 (19)	−0.0024 (19)

### Geometric parameters (Å, °)

**Table:**

S1—C5	1.710 (4)	C11—C12	1.382 (4)
S1—C2	1.729 (3)	C11—H11	0.9500
S2—C23	1.726 (4)	C12—C13	1.398 (5)
S2—C20	1.723 (3)	C12—H12	0.9500
O1—C7	1.233 (4)	C13—C14	1.396 (5)
O2—C18	1.230 (4)	C13—C16	1.458 (4)
C1—C2	1.497 (5)	C14—C15	1.378 (4)
C1—H1A	0.9800	C14—H14	0.9500
C1—H1B	0.9800	C15—H15	0.9500
C1—H1C	0.9800	C16—C17	1.325 (4)
C2—C3	1.348 (5)	C16—H16	0.9500
C3—C4	1.445 (5)	C17—C18	1.479 (5)
C3—H3	0.9500	C17—H17	0.9500
C4—C5	1.378 (5)	C18—C21	1.479 (5)
C4—C7	1.475 (5)	C19—C20	1.497 (4)
C5—C6	1.502 (5)	C19—H19A	0.9800
C6—H6A	0.9800	C19—H19B	0.9800
C6—H6B	0.9800	C19—H19C	0.9800
C6—H6C	0.9800	C20—C21	1.375 (4)
C7—C8	1.474 (5)	C21—C22	1.428 (5)
C8—C9	1.327 (5)	C22—C23	1.355 (5)
C8—H8	0.9500	C22—H22	0.9500
C9—C10	1.453 (4)	C23—C24	1.496 (5)
C9—H9	0.9500	C24—H24A	0.9800
C10—C11	1.386 (4)	C24—H24B	0.9800
C10—C15	1.407 (4)	C24—H24C	0.9800
			
C5—S1—C2	93.59 (17)	C13—C12—H12	119.6
C23—S2—C20	93.32 (16)	C14—C13—C12	117.7 (3)
C2—C1—H1A	109.5	C14—C13—C16	119.4 (3)
C2—C1—H1B	109.5	C12—C13—C16	122.8 (3)
H1A—C1—H1B	109.5	C15—C14—C13	121.7 (3)
C2—C1—H1C	109.5	C15—C14—H14	119.2
H1A—C1—H1C	109.5	C13—C14—H14	119.2
H1B—C1—H1C	109.5	C14—C15—C10	120.2 (3)
C3—C2—C1	128.9 (3)	C14—C15—H15	119.9
C3—C2—S1	109.7 (3)	C10—C15—H15	119.9
C1—C2—S1	121.4 (3)	C17—C16—C13	127.7 (3)
C2—C3—C4	114.5 (3)	C17—C16—H16	116.1
C2—C3—H3	122.7	C13—C16—H16	116.1
C4—C3—H3	122.7	C16—C17—C18	122.5 (3)
C5—C4—C3	111.3 (3)	C16—C17—H17	118.8
C5—C4—C7	123.6 (3)	C18—C17—H17	118.8
C3—C4—C7	125.0 (3)	O2—C18—C17	121.2 (3)
C4—C5—C6	129.9 (3)	O2—C18—C21	121.8 (3)
C4—C5—S1	110.9 (3)	C17—C18—C21	116.9 (3)
C6—C5—S1	119.3 (3)	C20—C19—H19A	109.5
C5—C6—H6A	109.5	C20—C19—H19B	109.5
C5—C6—H6B	109.5	H19A—C19—H19B	109.5
H6A—C6—H6B	109.5	C20—C19—H19C	109.5
C5—C6—H6C	109.5	H19A—C19—H19C	109.5
H6A—C6—H6C	109.5	H19B—C19—H19C	109.5
H6B—C6—H6C	109.5	C21—C20—C19	130.0 (3)
O1—C7—C8	120.6 (3)	C21—C20—S2	110.1 (3)
O1—C7—C4	120.9 (3)	C19—C20—S2	119.9 (3)
C8—C7—C4	118.4 (3)	C20—C21—C22	112.6 (3)
C9—C8—C7	122.1 (3)	C20—C21—C18	123.1 (3)
C9—C8—H8	118.9	C22—C21—C18	124.3 (3)
C7—C8—H8	118.9	C23—C22—C21	114.1 (3)
C8—C9—C10	127.5 (3)	C23—C22—H22	123.0
C8—C9—H9	116.2	C21—C22—H22	123.0
C10—C9—H9	116.2	C22—C23—C24	128.3 (3)
C11—C10—C15	118.3 (3)	C22—C23—S2	109.9 (3)
C11—C10—C9	120.0 (3)	C24—C23—S2	121.7 (3)
C15—C10—C9	121.7 (3)	C23—C24—H24A	109.5
C12—C11—C10	121.3 (3)	C23—C24—H24B	109.5
C12—C11—H11	119.4	H24A—C24—H24B	109.5
C10—C11—H11	119.4	C23—C24—H24C	109.5
C11—C12—C13	120.8 (3)	H24A—C24—H24C	109.5
C11—C12—H12	119.6	H24B—C24—H24C	109.5
			
C5—S1—C2—C3	0.4 (3)	C12—C13—C14—C15	0.2 (5)
C5—S1—C2—C1	−179.9 (3)	C16—C13—C14—C15	−178.9 (3)
C1—C2—C3—C4	−179.1 (3)	C13—C14—C15—C10	0.0 (5)
S1—C2—C3—C4	0.6 (4)	C11—C10—C15—C14	−0.6 (5)
C2—C3—C4—C5	−1.6 (5)	C9—C10—C15—C14	180.0 (3)
C2—C3—C4—C7	176.2 (3)	C14—C13—C16—C17	−173.7 (3)
C3—C4—C5—C6	−177.4 (4)	C12—C13—C16—C17	7.2 (6)
C7—C4—C5—C6	4.8 (6)	C13—C16—C17—C18	−179.8 (3)
C3—C4—C5—S1	1.8 (4)	C16—C17—C18—O2	−4.4 (5)
C7—C4—C5—S1	−176.0 (3)	C16—C17—C18—C21	174.5 (3)
C2—S1—C5—C4	−1.3 (3)	C23—S2—C20—C21	−1.1 (3)
C2—S1—C5—C6	178.0 (3)	C23—S2—C20—C19	−179.8 (3)
C5—C4—C7—O1	0.0 (5)	C19—C20—C21—C22	178.7 (3)
C3—C4—C7—O1	−177.6 (3)	S2—C20—C21—C22	0.1 (4)
C5—C4—C7—C8	176.5 (3)	C19—C20—C21—C18	0.6 (6)
C3—C4—C7—C8	−1.0 (5)	S2—C20—C21—C18	−178.0 (3)
O1—C7—C8—C9	9.4 (5)	O2—C18—C21—C20	−24.7 (5)
C4—C7—C8—C9	−167.2 (3)	C17—C18—C21—C20	156.4 (3)
C7—C8—C9—C10	178.5 (3)	O2—C18—C21—C22	157.4 (3)
C8—C9—C10—C11	171.8 (3)	C17—C18—C21—C22	−21.5 (5)
C8—C9—C10—C15	−8.8 (6)	C20—C21—C22—C23	1.3 (4)
C15—C10—C11—C12	1.0 (5)	C18—C21—C22—C23	179.3 (3)
C9—C10—C11—C12	−179.6 (3)	C21—C22—C23—C24	178.6 (3)
C10—C11—C12—C13	−0.8 (5)	C21—C22—C23—S2	−2.0 (4)
C11—C12—C13—C14	0.2 (5)	C20—S2—C23—C22	1.8 (3)
C11—C12—C13—C16	179.2 (3)	C20—S2—C23—C24	−178.8 (3)
